# WebNetCoffee: a web-based application to identify functionally conserved proteins from Multiple PPI networks

**DOI:** 10.1186/s12859-018-2443-4

**Published:** 2018-11-12

**Authors:** Jialu Hu, Yiqun Gao, Junhao He, Yan Zheng, Xuequn Shang

**Affiliations:** 1 School of Computer Science, Northwestern Polytechnical University, Xi’an, 710072 China; 2 Centre for Multidisciplinary Convergence Computing, School of Computer Science, Northwestern Polytechnical University, Xi’an, 710129 China

**Keywords:** Multiple network alignment, Webserver, PPI networks, Protein databases, Gene ontology

## Abstract

**Background:**

The discovery of functionally conserved proteins is a tough and important task in system biology. Global network alignment provides a systematic framework to search for these proteins from multiple protein-protein interaction (PPI) networks. Although there exist many web servers for network alignment, no one allows to perform global multiple network alignment tasks on users’ test datasets.

**Results:**

Here, we developed a web server WebNetcoffee based on the algorithm of NetCoffee to search for a global network alignment from multiple networks. To build a series of online test datasets, we manually collected 218,339 proteins, 4,009,541 interactions and many other associated protein annotations from several public databases. All these datasets and alignment results are available for download, which can support users to perform algorithm comparison and downstream analyses.

**Conclusion:**

WebNetCoffee provides a versatile, interactive and user-friendly interface for easily running alignment tasks on both online datasets and users’ test datasets, managing submitted jobs and visualizing the alignment results through a web browser. Additionally, our web server also facilitates graphical visualization of induced subnetworks for a given protein and its neighborhood. To the best of our knowledge, it is the first web server that facilitates the performing of global alignment for multiple PPI networks.

**Availability:**

http://www.nwpu-bioinformatics.com/WebNetCoffee

## Background

Proteins are involved in almost all life processes. The discovering of protein function is of significance for understanding the underlying molecular mechanism in organisms and diseases. Thanks to the development of high-throughput technologies, computational approaches become a major force to uncover molecular structures and functions [1, 2]. Since then, a large amount to genomics, proteins, interactions, pathways and functional annotation data have been accumulated and freely available in public databases, such as Genbank [3], Uniprot [4], KEGG [5] and RSCB PDB [6]. So far, many computational tools [7–10] have been developed to understand molecular function by using genomic sequences, pathways and molecular networks. However, there is still a large gap to obtain a comprehensive knowledge of protein function for various species [11].

Global network alignment is an efficient framework to systematically identify functionally conserved proteins from different species. It aims to search for an optimal global node map for all nodes in different PPI networks. These proteins matched in one group are thought to be functionally conserved, which are also called function-oriented ortholog (FO) groups. Therefore, one can predict the function of an uncharacterized protein according to the functional annotation of another protein in its FO group.

The results of a global network alignment consist of a series of matchsets for two or more PPI networks. Each matchset of the node map represents a putative FO group. IsoRank [12] was firstly developed to identify FO groups between two given PPI networks. The alignment was intuitively guided by an idea that two proteins matched if and only if their neighbors can also be well matched. The algorithm was later improved in IsoRankN [13] to find a global alignment for multiple networks (GAMN). IsoRankN takes spectral partitioning method to find alignment clusters on the induced graphs of pairwise alignment scores. Since then, a bunch of multiple global alignment tools have been developed one after another, most notably Graemlin 2.0 [14], SMETANA [15], NETAL [16], NetCoffee [17], MAGNA [18] and MAGNA++ [19].

However, most of these alignment tools depend on some existing libraries, environment configuration or many input datasets with a particularly designed format, which is difficult to prepare. And the command line interface (CLI) of these computational tools cannot provide a graphical user interface to manage the data, visualize the processing status and annotate the alignment results using public databases. Several web servers have been developed to perform local alignments, to query pathways and patterns, including NetworkBlast [20], PINALOG [21], NetAligner [22], PathBlast [23], NetalieQ [24], etc. However, as far as we know, very few of web servers facilitate the aligning of multiple PPI networks. To make the task of GAMN easier to be done for non-expert users, here, we present a web server WebNetCoffee based on the NetCoffee algorithm, which can fast and accurately search for a global node map for multiple PPI networks. The web server is available at http://www.nwpu-bioinformatics.com/WebNetCoffee/, which enables users to upload their own test datasets or select three or more species from four well-known PPI databases IntAct [25], STRING [26], DIP [27] and BioGRID [28].

## Implementation

We implemented the web server WebNetCoffee in several programming languages, which include C++, html, php, css and mysql. The graph library LEMON version 1.2.3 [29] was used in the implementation of NetCoffee. The Apache HTTP Server and MySQL provide the fundamental web server environment. The server runs on a CPU of Intel(R) Xeon(R) CPU E5-2682 v4 @ 2.50GHz.

### The workflow of NetCoffee

Given a set of networks *G*_1_,*G*_2_,⋯,*G*_*k*_, *k*≥3, each network can be modeled as a graph *G*_*i*_=(*V*_*i*_,*E*_*i*_), where *V*_*i*_ and *E*_*i*_ represent proteins and interactions appearing in networks. Proteins aligned in one group is a matchset, which is a subset of $\cup _{i=1}^{k}V_{i}$. The global network alignment problem is to search for a set of mutually disjoint matchsets for two or more PPI networks. We assumed that the sequence similarity and topology similarity can imply the functional conservation of proteins in different species.

An integrated model was adopt to measure the similarity of a given pair of nodes by using both topology and sequence information in the NetCoffee algorithm [17]. It employs simulated annealing to optimize a target function, aiming to search for an optimal global one-to-one map based on similarity of both network topology and protein sequences. There are four major steps: 1) building PPI networks and a library of bipartite graphs; 2) calculating integrated weight using triplet extension; 3) collecting candidate edges with maximum matching; 4) optimization with the simulated annealing approach. NetCoffee was distinguished itself with other existing algorithms by its fast speed and biologically meaningful alignment. It can perform a GAMN job on three or more PPI networks. The alignment result consists of a lot of matchsets, each of which represents a putative functional ortholog group.

### Using WebNetCoffee

The WebNetCoffee provides a simple web interface for performing GAMN tasks. The home page of WebNetCoffee briefly introduces the foundation of NetCoffee and the resources of online datasets. The help page can quickly guide a new user to perform a GAMN task and query the results through a jobid step by step. Besides, it also gives more detailed description for each panel in the result page.

**Fig. 1:**
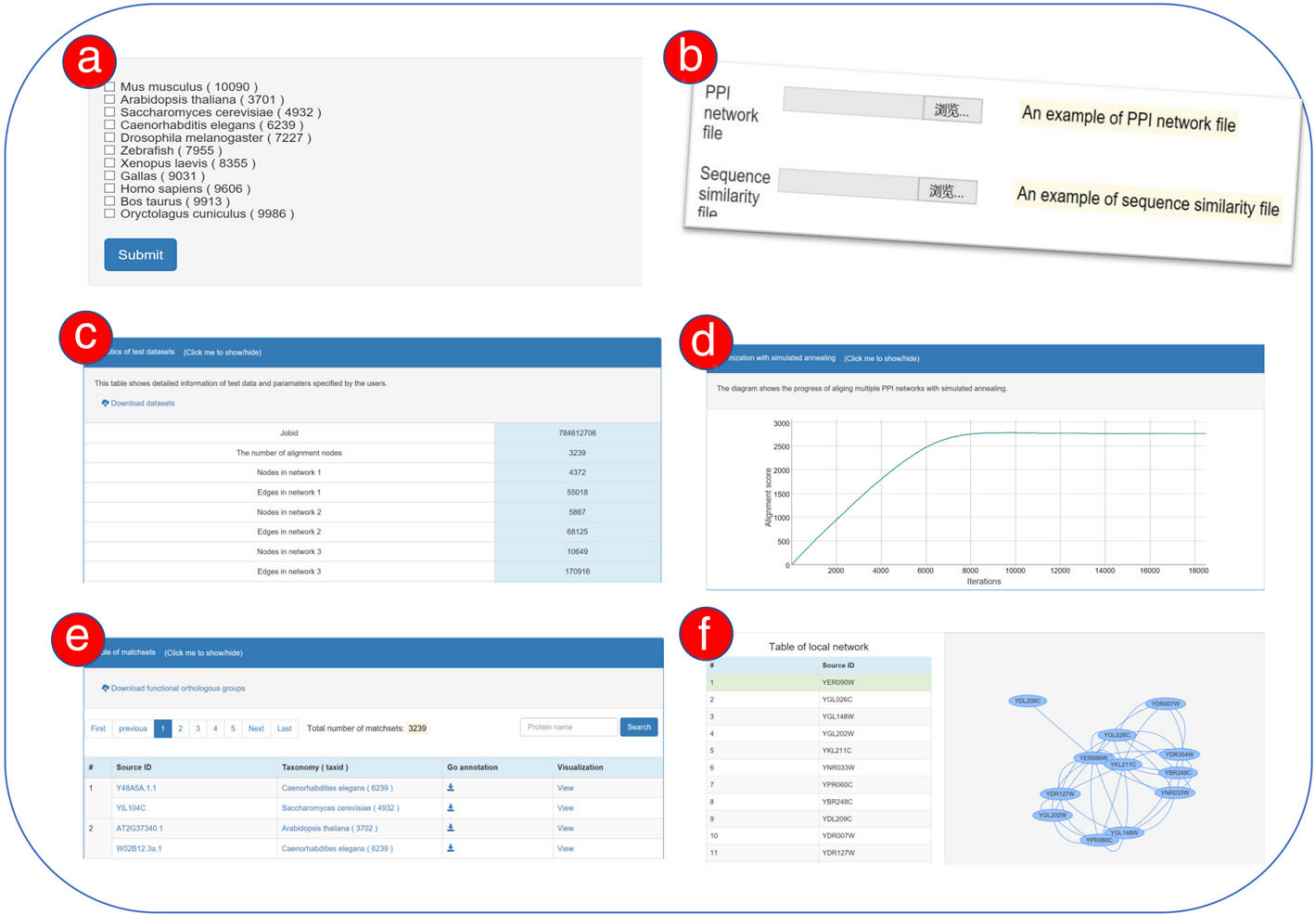
A workflow of WebNetCoffee. **a** Networks of up to 15 species are available for users’ options, which were manually collected from four commonly used databases. **b** Users can perform WebNetCoffee on their own datasets. **c** Statistics of test datasets. **d** Convergence curves of simulated annealing. **e** Table of the functionally conserved protein groups. **f** Visualization of induced sub-networks

Users can launch a WebNetCoffee job on both the online datasets and users’ own datasets (see in Fig. 1a,b). Each job can be assigned a user-specified job title. The default parameter of alpha is 0.5, which is used to balance the contribution of topology and sequence score in the alignment result. To launch a job on the online datasets, totally, 15 species from four databases are available for users’ options. One can choose three or more species for each GAMN task. To avoid users’ very large computational tasks, WebNetCoffee allows performing a job on 3-5 networks in BioGRID and STRING, 3-11 networks in IntAct and DIP. Each file of users’ datasets uploaded to the server is restricted to be less than 200M.

In case many tasks were submitted to the server simultaneously, we designed a job queue to manage all the jobs. Each submitted job would firstly go into the job queue, waiting for a time slot in the server. There is a watchdog managing the submitted jobs with the principle of first come first serve (FCFS). It checks the status of the job queue in the background at regular intervals. The earliest job will start to run when a time slot was assigned to it. Users can query their results through a jobid within one week after the job finished. In one week, it will expire automatically. For the protection of privacy, each user can only see these jobs submitted by themselves (with the same IP address) in the job list. It also allows users to set a password before launching a job, which can avoid privacy leaks when multiple users shares a same IP address.

In the result page, we present statistics of test datasets in the first part, which include nodes and edges of each input network, the final alignment score and input parameters etc (see in Fig. 1c). To visualize the process of simulated annealing, the convergence curve is plotted in the second part. From Fig. 1d, users can see how fast the computation can converge to a stable score. In the third part, there is a large table separated in many pages, each page contains at most ten matchsets (see in Fig. 1e). Each matchset implies a group of functionally conserved proteins, which can be used in the “annotation transfer”. Additionally, our web server can provide information from open accessible databases to annotate the alignment results, such as the Uniprot ID, and gene ontology annotations. Each protein is linked to its GenPept page in the open accessible database NCBI protein. Users can easily check their sequence similarity and download function annotations (GO terms) by a simple click. Besides, users can also search for specific matchsets with a pattern in a search box. For example, if a substring “P535” was queried in the search box, the protein accession identifiers matched to the pattern “%P355%” will be extracted from the result table. In the column of visualization, the graphical view of induced sub-networks would be extracted from its PPI networks (see in Fig. 1f). The test dataset and alignment results of each task are available in the result page, which makes it very easy to run other methods on our online datasets and to compare the alignment quality.

## Results

**Fig. 2:**
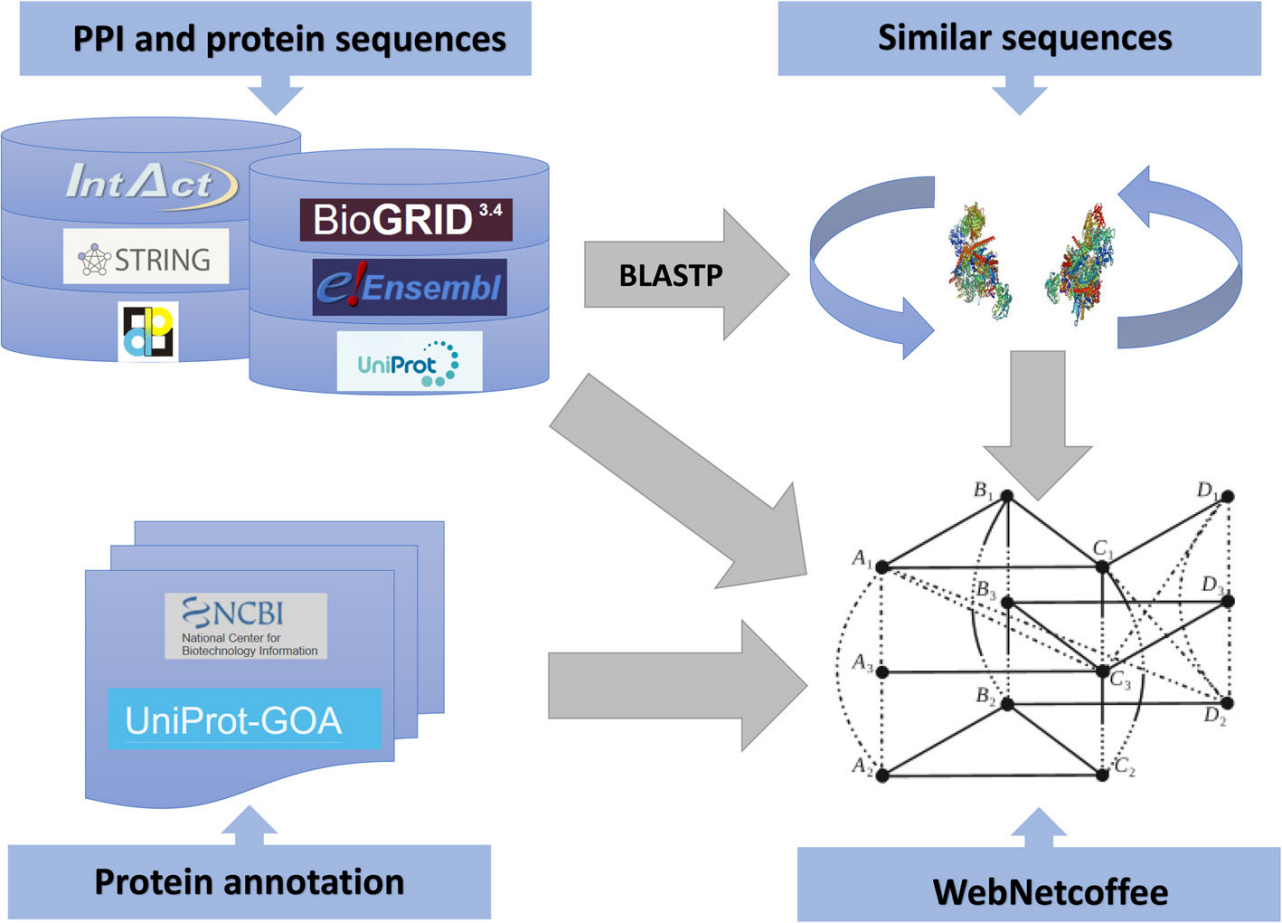
Overview of the WebNetCoffee resources. We manually collected our testing datasets from eight openly accessible databases for up to 15 species. Protein-protein interactions (PPIs) and protein sequences were extracted from IntAct, STRING, DIP, BioGRID, UniProt, and Ensembl. Protein annotation data such as gi number and gene ontology annotations were extracted from the NCBI Protein and Uniprot-GOA project. Using the BLAST package, we performed pairwise sequence alignments to search for similar protein sequences

**Fig. 3:**
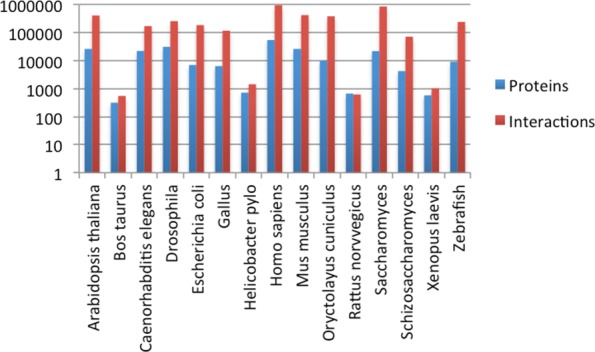
Statistics of proteins and molecular interactions in our online datasets. All these 215,002 proteins and 4,005,485 interactions of 15 species are openly accessible to researchers

As network topology and protein sequences were required for scoring the alignment, we manually collected a set of data from a series of openly accessible online resources storing protein-protein interactions, protein sequences, and protein annotation data (see in Fig. 2). Totally, 218,339 proteins and 4,009,541 interactions were extracted from six freely available databases, including IntAct, STRING, DIP, BioGRID, UniprotKB [30] and Ensembl [31] (see details in Fig. 3). Since proteins in different sources labeled by different identifiers, we convert all different sources identifiers into Uniprot identifier in the format acession:version, which is commonly used in many famous databases such as IntAct, QuikGO [32], UniProtKB/Swiss-Prot [33] and the NCBI protein databases [34]. This identifiers were further used to query the GO annotation in our web tools.

Proteins of up to 15 species and their associated annotation data including taxonomy information, gene ontology were collected to build our online datasets. To improve the data quality, interactions generated by co-complex expansion (in IntAct) and these with a small combined score (less than 900) in STRING were filtered away. The package of BLASTP [35] was performed to calculate the pairwise sequence similarity. These pairs of homologous proteins with sufficient common region (evalue <1*e*−7) are likely to be in a same matchset. Our online datasets including interactions and homologous proteins are openly accessible to researchers for performance comparison in the Download page. All our online datasets are regularly updated once every three months if there exist latest updates in the corresponding databases. Hopefully, it can promote the development of more advanced network alignment tools, facilitate the build of benchmark datasets, and lead us to a better understanding of molecular evolution and functions of these uncharacterized proteins.

## Conclusion

In this paper, we present a fast and versatile web server based on the network alignment algorithm of NetCoffee to search for functional orthologous groups from multiple PPI networks. To the best of our knowledge, it is the first web server for globally aligning multiple PPI networks. Compared to NetCoffee, it also provides a friendly graphical user interface for easily performing GAMN tasks, managing submitted jobs and visualizing the alignment results. In the following work, we will support network alignment of multiple networks from more commonly used databases, and keep WebNetCoffee up-to-date with the latest data from these databases. Furthermore, the algorithm will be extended to take into account GO terms and pathways, which is expected to improve the prediction accuracy and facilitate more advanced applications such as prediction of disease-related genes.

## Availability and requirements

**Project name:** WebNetcoffee

**Project home page:** http://www.nwpu-bioinformatics.com/WebNetCoffee/

**Operating system(s):** Platform independent

**Programming language:** C++, html, css, PHP

**Other requirements:** None

**License:** GNU GPL v3

**Any restrictions to use by non-academics:** license needed

## Abbreviations

CLI: Command line interface
FO: Function-oriented ortholog
FCFS: First come first serve
GAMN: Global alignment for multiple networks
GO: Gene ontology
PPI: Protein-protein interactions
